# 20th International Conference on Emerging Infectious Diseases in the Pacific Rim Organized by the United States-Japan Cooperative Medical Sciences Program (USJCMSP)

**DOI:** 10.3390/vaccines7020035

**Published:** 2019-04-02

**Authors:** USJCMSP AIDS, ARI, Cancer, Hepatitis, Immunology and Viral Diseases Panels, K. Gayle Bernabe, Kristina T. Lu, F. Gray Handley, Diane E. Griffin, Ichiro Kurane, Aikichi Iwamoto, George F. Gao, Florian Krammer

**Affiliations:** 1 Department of Microbiology, Icahn School of Medicine at Mount Sinai, New York, NY 10029, USA; 2 Office of Global Research, NIAID, NIH, Bethesda, MD 20892, USA; 3 Division of Microbiology and Infectious Diseases, NIAID, NIH, Bethesda, MD 20892, USA; lukr@mail.nih.gov; 4 Office of the Director, NIAID, NIH, Bethesda, MD 20892, USA; handleygr@niaid.nih.gov; 5 Department of Molecular Microbiology and Immunology, Johns Hopkins Bloomberg School of Public Health, Baltimore, MD 21205, USA; dgriffi6@jhmi.edu; 6 National Institute of Infectious Diseases, Shinjuku-ku, Tokyo 162-8640, Japan; kurane@niid.go.jp; 7 Department of Research Promotion, Japan Agency for Medical Research and Development, Chiyoda-ku, Tokyo 100-0004, Japan; aikichi@zj9.so-net.ne.jp; 8 CAS Key Laboratory of Pathogenic Microbiology and Immunology, Chinese Academy of Sciences Institute of Microbiology, Beijing 100101, China; gaof@im.ac.cn

**Keywords:** emerging infectious diseases, pacific rim

## Abstract

The 20th International Conference on Emerging Infectious Diseases in the Pacific Rim to3ok place in Shenzhen, China on January 8–9, 2018 followed by meetings of the acquired immunodeficiency syndrome (AIDS)/immunology, acute respiratory infections, cancer, hepatitis, and viral diseases panels on January 10–11. The conference was organized as part of the United States-Japan Cooperative Medical Sciences Program (USJCMSP) by the Japan Agency for Medical Research and Development (AMED) and the U.S. National Institutes of Health (NIH) and was locally hosted by the Shenzhen Third People’s Hospital and the Chinese Academy of Sciences (CAS) Institute of Microbiology. The conference provides the basis for networking and fostering of collaboration opportunities between researchers in Southeast Asia and the United States based on the scientific and interactive platform of the USJCMSP and takes place in the region on an annual basis. This report summarizes the discussions and conclusions from the conference.

## 1. Introduction

The United States-Japan Cooperative Medical Sciences Program (USJCMSP) was formed under a joint communiqué issued by U.S. President Lyndon B. Johnson and Japanese Prime Minister Eisaku Sato in 1965 to expand the cooperative research effort in the medical sciences and focus on the biomedical needs of Asian countries in the Pacific Rim [1]. USJCMSP initially concentrated on cholera, leprosy, parasitic diseases, tuberculosis, and viral diseases, with each of these areas forming a panel that included U.S. and Japanese scientists [2,3,4,5,6,7,8]. This focus has evolved over time to reflect changing public health issues in the region and the current nine panels concentrate on 1) acute respiratory infections (ARI); 2) acquired immunodeficiency syndrome (AIDS); 3) cancer; 4) cholera and other bacterial enteric infections; 5) hepatitis; 6) mycobacterial diseases; 7) nutrition and metabolism; 8) parasitic diseases; and 9) viral diseases. A separate cross-cutting board focuses on immunology.

In 1996, the USJCMSP launched a conference series called the “International Conference on Emerging Infectious Diseases (EID) in the Pacific Rim” that is typically held annually in different countries in the region (Table 1). During these conferences, participants exchange information, ideas, and share current research on varying diseases of importance in the Asia-Pacific region, with the intent to foster research collaborations. Following the EID conference, the specific panels/board hold one–two-day meetings. The focus of the conference alternates yearly between viral diseases and bacterial and parasitic diseases. 2018 marked the 20th anniversary of this conference series. The 20th International Conference on EID in the Pacific Rim was held on 8–9 January, 2018 and was followed by panel/board meetings on January 10–11, 2018. The conference took place at the Coli Hotel in Shenzhen, People’s Republic of China, and was locally hosted by the Shenzhen Third People’s Hospital and the Chinese Academy of Sciences (CAS) Institute of Microbiology. This report summarizes the presentations (including the latest progress and gaps from the various fields of research), discussion, and conclusions of the conference.

## 2. 2018 EID Conference

The conference began with two lectureships in honor of four longstanding champions of the USJCMSP: Dr. John Ring La Montagne (deceased), Dr. Carole A. Heilman, Dr. Tadao Shimao, and Dr. Yoshifumi Takeda.

**Figure 1:**
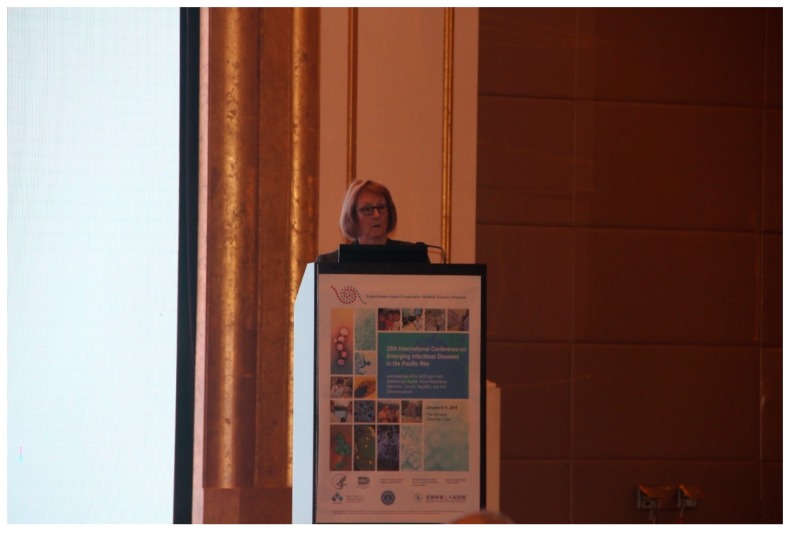
Dr. Mary Estes—La Montagne-Heilman Lectureship (Photo used with permission from Dr. Estes).

**Figure 2:**
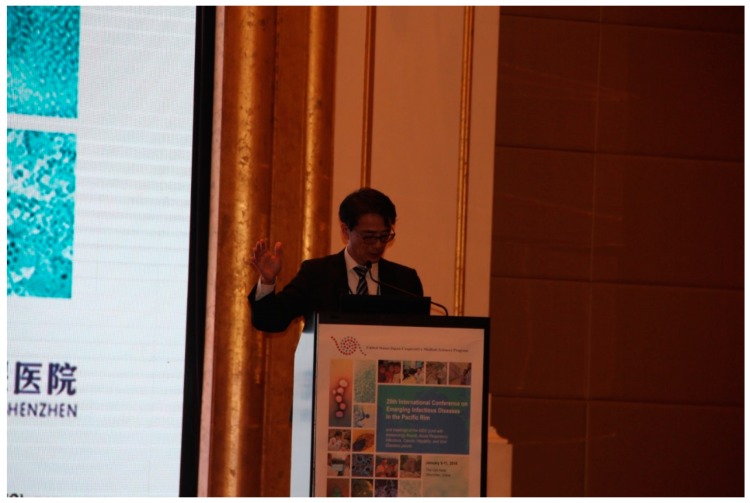
Dr. Takaji Wakita—Shimao-Takeda Lectureship (Photo used with permission from Dr. Wakita).

a) La Montagne-Heilman Lectureship—Dr. Mary K. Estes (Baylor College of Medicine, U.S.A.) presented a talk entitled “Translating stem cell biology to understand gastrointestinal virus infection” (Figure 1). She discussed the utilization of human intestinal enteroid (HIE) cultures as pre-clinical models to study the response of the intestinal epithelium to human gastrointestinal infections, such as rotavirus and norovirus. HIEs could lead to a better understanding of human host-pathogen interactions, including innate immune responses, and potentially to identifying and testing new drug therapies for the prevention and treatment of diarrheal diseases.
b) Shimao-Takeda Lectureship—Dr. Takaji Wakita (National Institute of Infectious Diseases, Japan) presented a talk, “Optimization of hepatitis C virus treatment and post-SVR syndrome” (Figure 2). Dr. Wakita discussed the development of a method that combines cell culture data with a mathematical model and computer simulation to quantify the anti-hepatitis C virus (HCV) efficacy of different drug concentrations and combinations in a preclinical setting. He reported that HCV treatment with direct-acting antivirals could achieve a sustained virologic response (SVR). However, a significant number of patients who achieve SVR developed hepatocellular carcinoma. Abnormal hepatocellular organelles in SVR patients indicate a persistent post-SVR syndrome.

### 2.1. Flash Talks from Recipients of the 2016 USJCMSP Collaborative Awards

The USJCMSP Collaborative Awards initiative, established following the 50th anniversary of the program in 2016, supports new or expanded infectious disease and immunology research collaborations between researchers in Japan, the Asia-Pacific region, and the United States. The initiative also promotes and facilitates the inclusion of early-stage and female investigators in collaborative research in the region. The EID conference provided a platform for award recipients to highlight their collaborative research through brief talks and poster presentations.

### 2.2. Plenary Sessions

The EID conference featured five plenary sessions focused on the pathogenesis and protective immunity of viral diseases that are of priority in the region and have global health importance.

#### 2.2.1. Session 1

Session 1 focused on the recent trends of viral infections in Asia and the Pacific Rim. This session aimed to review some of the important new, long-time, and re-emerging infectious viral threats in Asia, and to consider the research and public health response needed for addressing them. Information was provided about newly recognized viral infections (e.g., Crimean–Congo hemorrhagic fever virus (CCHFV)) and previously under-appreciated epidemiology of well-known viral infections (e.g., hepatitis viruses), as well as highlights of serious virus outbreaks currently in the public eye (e.g., dengue and Zika).

- Avian influenza: The first presentation focused on the threat of avian influenza A H7N9 virus, which currently causes zoonotic infections that are mostly acquired in live bird markets. One rest day (closure) in poultry markets has a significant impact in reducing virus persistence and transmission of the virus through aerosols in poultry handling and de-feathering areas. The reported cases of H7N9 influenza underestimates the extent of human infection, and the effect of vaccination needs to be further studied.
- Hepatitis: Another talk reported on the endemic prevalence of hepatitis viruses in Mongolia, which has the highest rate of liver cancer mortality in the world that is about 12 times the global average. The high prevalence is mostly health-system driven, with the use of dirty needles and improper sterilization of dental equipment, but injection drug abuse also contributes. Sixty percent of hepatitis B virus (HBV)-infected Mongols are co-infected with hepatitis delta virus (HDV). The government of Mongolia adopted a plan that includes screening every citizen for hepatitis virus infection to prevent, control, and eventually eliminate hepatitis in Mongolia.
- Dengue: An overview of dengue, a public health priority in the region, was presented. The distribution of dengue is widespread and inflicts a high medical and cost burden. The pathogenesis of dengue virus infection remains unclear, and there are pitfalls in diagnosis and management of the disease. There has been a shift in the epidemiology, with infections and disease moving to rural areas.
- Zika: A presentation of the current state of Zika virus (ZIKV) epidemiology was provided. ZIKV is amplified much more frequently than other viruses through *Aedes aegypti* and *Aedes albopictus* mosquitos. Particularly in Africa, strains were seen to be transmitted much more efficiently by *Aedes aegypti* and there is no evidence that primates are intermediates of transmission. Several different topics were discussed about ZIKV including the association between ZIKV infection and microcephaly and Guillain-Barre syndrome, when and how ZIKV arrived in the Americas, and whether chikungunya and dengue virus infections can provide insight into ZIKV infection in the Americas. Several studies have failed to provide evidence of immune enhancement, although the question remains if dengue virus immunity affects ZIKV infection or vice versa. The evolution of urban transmission was also considered, with the emergence of different virus strains.
- Crimean–Congo hemorrhagic fever (CCHF): The expansion of CCHF to Asia was reviewed. CCHFV is an orthonairovirus transmitted by the bite of the tick, *Hyalomma marginatum*. The incubation period of the infection is 2–7 days, and death occurs in 7–9 days. The re-assortment of genes among the CCHF viruses can lead to new strains and new diseases, with many countries reporting increased numbers of cases. This may be due to more infected ticks, amplification, migratory birds, or the live animal trade. Increased tick activity has been observed during the Eid festival, which coincides with backyard slaughter of animals, and an increase in human infection.

#### 2.2.2. Session 2

Session 2 focused on the pathogenesis of emerging viral infections affecting Southeast Asian countries and their divergent mechanisms.

- Hepatitis C: The first talk in this session compared the level of immune markers, such as PD-1 and CD91, before and after antiviral treatment for hepatitis C virus (HCV) infection and found that those levels were lower after treatment.
- Coronavirus: The next presentation shared how in coronaviruses, exoribonuclease (ExoN) plays a key role in maintaining a low mutational rate for the virus, and exon deletion increases the mutation rate as well as the attenuation of pathogenesis.
- Enterovirus 71 (EV71): Then a talk focused on the EV71 carrying VP1-145E variants, which are mainly responsible for the development of viremia and neuropathogenesis in a non-human primate model. This further suggested the involvement of amino acid polymorphism at VP1-145 in cell-specific viral replication, in vivo fitness, and pathogenesis in EV71-infected individuals.
- HIV-1: One talk presented that HIV-1 integrase (IN) is a key target of newly developed anti-retroviral drugs. High-resolution cryo-EM analysis demonstrated that IN assembles into a multimeric structure, up to 16 monomers in size, to form the intasome structure that mediates chromosomal integration. The resolution of the active site revealed the strand transfer inhibitor drug target, as well as potential new structural targets for next-generation IN inhibitors. Another talk reported that human leukocyte antigen (HLA)-B57 and HLA-B27 haplotypes are associated with the control of HIV-1 viremia and disease progression in Caucasian and African populations, but are poorly represented in people of Japanese descent. The research team found that HLA-B52:01 and HLA-C12:02, found in approximately 20% of Japanese individuals, is associated with significantly lower HIV-1 viral loads and increased CD4 cell counts. In addition, the natural killer (NK) cell receptor, killer cell immunoglobulin-like receptor (KIR) 2DL2, may also contribute to the control of HIV-1 in Japanese individuals, suggesting both cytotoxic T lymphocyte (CTL) and NK responses play an important role in controlling HIV disease progression.
- Reovirus: A talk featured the reovirus reverse genetic system, which was established by transfecting the cell via plasmids, each coding a segment of reovirus genome. This is a major development for the reovirus field.
- Hepatitis B (HBV): The host Na^+^-taurocholate cotransporting polypeptide (NTCP) protein, found to be an essential component for HBV infection in the liver, was discussed. The Ser267Phe (S267F) variant of NTCP is inversely associated with HBV pathogenesis and cirrhosis. The NTCP sequence is also a determinant for host range.
- Zika: A talk showed that dengue 2 antibody levels appear to determine susceptibility to ZIKV infection with high dengue 2 titers being protective, while lower titers enhance infection.

**Figure 3:**
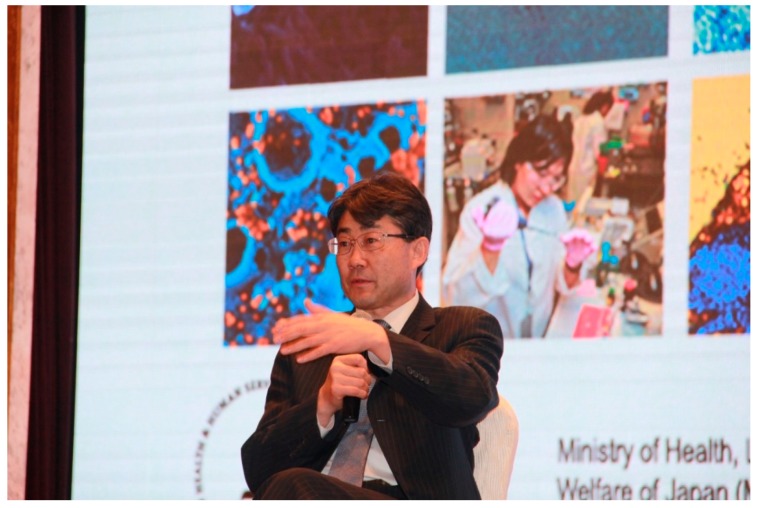
Local host Dr. George F. Gao (Photo used with permission from Dr. Gao).

Infectious Disease Control—The final talk for the session by Dr. George Fu Gao (Figure 3) highlighted the key elements of China’s 65 years of experience in infectious disease control through its Patriotic Health Campaign, which includes government commitment and public education. This infectious disease control approach could potentially be applied in other parts of the world.

#### 2.2.3. Session 3

The session on hepatitis virus infection and liver cancer provided a research update on: the molecular virology and immunology of HBV; host factors related to hepatitis virus infection; the genetic landscape of virus-associated hepatocellular carcinoma (HCC); integrated genomics to identify drivers of human liver cancers; chemical-viral interaction between aflatoxin and HBV in induction of HCC; and antibody therapeutics targeting glypican-3 (GPC3) for the treatment of liver cancer. Through an extensive clinical network of patients in China, randomized clinical trials are being conducted to study new treatment strategies for chronic HBV infection involving nucleotide analog reverse transcriptase inhibitors and peginterferon. An ongoing effort to prevent mother–child transmission of HBV through a short-term antiviral therapy with tenofovir disoproxil fumarate during late pregnancy, reported a significantly lower HBV transmission rate compared to the control group. This session also included a discussion of host factors related to hepatitis virus infection using a genome-wide association study (GWAS). A genetic analysis using GWAS, identified host factors for various human multifactorial diseases, as well as interferon (IFN) lambda for drug response, and HLA II genes for susceptibility to chronic HBV infection (CHB). Frequencies of HLA-DP risk alleles are high in Asian populations, whereas frequencies of HLA-DP protective alleles are high in European populations. These findings could explain the high incidence of CHB in Asian countries and suggest that host genetic factors are important to viral infections. Another talk outlined the genomic and epigenomic associations in HBV-related liver cancer using data obtained from whole genome bisulfate sequencing and whole genome sequencing. Clonal HBV integrations preferentially occurred in inactive chromatin regions; massive rearrangements were detected in the integrated HBV genome, and a negative correlation exists between HBV rearrangement number and total somatic mutation number. These observations could be useful for understanding the progression of HBV-related liver cancer. It was noted that liver cancer, including HCC and cholangiocarcinoma, is the second leading cause of cancer death (about 9.1% of total cancer deaths) with a significant burden in low- and middle-income countries in Asia and sub-Saharan Africa. Etiological factors associated with HCC include infection with HBV and exposure to high levels of aflatoxin B1 (AFB1) in the diet. HCV-associated HCC is becoming the most rapidly rising solid tumor in the United States and Japan. The development of highly effective drugs that cure HCV infection is a major advance that, hopefully, will diminish the role of HCV in liver cancer. Ongoing clinical investigations are defining the utility of GPC3, a cell surface proteoglycan differentially expressed in HCC, and other promising antibody therapeutics to treat liver cancer.

#### 2.2.4. Session 4

Session 4 focused on vaccines, with seven presentations on the current state and future landscape of vaccine development, technology, promising candidates, and clinical outcomes for prominent viral infectious diseases: Dengue, ZIKV, respiratory syncytial virus (RSV) infection, HIV/AIDS, influenza virus, hand-foot-and-mouth disease (HFMD), and chikungunya.

- Dengue: A description of the dengue vaccine target product profile and an overview of an experimental live-attenuated dengue vaccine developed primarily by National Institute of Allergy and Infectious Diseases (NIAID), which was found to be safe and protective in virus challenge studies, were provided. An ongoing Phase 3 clinical trial in Brazil was also discussed.
- Zika: An overview of a promising experimental single-dose live-attenuated Zika vaccine that was evaluated in non-human primates was presented. This was found to prevent infection and achieve protective immunity within 14 days post-vaccination.
- Respiratory syncytial virus (RSV) infection: The global disease burden of RSV and the need for more than one RSV vaccine to target different age populations were described. Also an overview of the RSV vaccines currently in clinical development: vaccine-like particle, live-attenuated, and vector-based vaccines, were further discussed.
- HIV/AIDS: A talk summarized the findings from a cohort study of individuals identified with acute HIV infection in Bangkok, Thailand.
- Influenza: A detailed description of a universal influenza virus vaccine strategy based on the conserved stalk domain of hemagglutinin was presented.
- Hand-foot-and-mouth disease (HFMD): The epidemiological baseline of HFMD caused by multiple serotypes of enterovirus in China and the implications for vaccine development were discussed.
- Chikungunya: A presentation described the versatility and advantages of a measles vector vaccine platform and the Phase 2 clinical safety and immunogenicity of a measles-vectored chikungunya vaccine.

The question and answer sessions focused on identification of correlates of protection; evaluation of vaccine candidates in immunocompromised populations; antibody enhancements; and the stability of attenuation in vaccine strains.

#### 2.2.5. Session 5

The last plenary session included three presentations that addressed new approaches to define protective immunity and drug discovery.

- Norovirus: A high-resolution structural analysis of viral proteins has provided a platform for a rational drug design for norovirus. With a better understanding of innate immunity, this could lead to new approaches for vaccine delivery. A new protein delivery system was also introduced, which may potentially become a component of a needleless vaccine candidate.
- ZIKV: The structural biology of ZIKV proteins suggests potential drug targets in the non-structural 1 (NS1) and non-structural 5 (NS5) viral proteins. The NS1 structure could provide information on host interaction and protective antibody recognition, as well as potential drug target sites. Structural analysis needs to be confirmed with functional data to demonstrate further if drug candidates, such as those against dengue virus, would be effective for ZIKV infection.

Toll-like receptor 7 (TLR7): The mechanisms controlling innate immune responses to nucleic acids were presented. The pathophysiological roles of nucleotide-sensing by TLR7 in inflammatory disorders, and possible ways that TLR7 might induce antiviral immune responses were discussed.

## 3. Panel/Board Meetings

After the plenary sessions of the larger EID conference, five concurrent meetings of the USJCMSP AIDS panel with the immunology board, acute respiratory infectious panel, cancer panel, hepatitis panel, and viral diseases panel convened to focus on specific topics relevant to each of the panels/boards.

### 3.1. AIDS Panel and Immunology Board

The AIDS panel and immunology board convened a joint meeting on 10–11 January, 2018. It was co-chaired by Drs. Thomas Hope (Northwestern University, U.S.A.) and Tetsuro Matano (University of Tokyo/National Institute of Infectious Diseases, Japan), and secretariat Dr. David McDonald (NIAID, U.S.A.). Dr. Maureen Goodenow (Director of the NIH Office of AIDS Research, U.S.A.) opened the meeting with an overview of AIDS research funding at the NIH and emphasized the importance of basic research in support of clinical approaches for prevention, treatment, and cure of HIV/AIDS. Session 1 on the “Clinical Impact of HIV in the Asia-Pacific Region” featured reports of HIV/AIDS in Mongolia, Vietnam, and Thailand. Session 2 on “HIV Molecular Virology” featured talks focused on the cell and molecular biology of HIV. Session 3 on “HIV, Host Cell, and Microbiome Interactions” included presentations about host cell factors that impact HIV assembly, entry and immune responses, as well as a potential new source of latently infected cells in infected individuals. The final session titled “Mucosal Immunology and Vaccine Responses” included discussions of mucosal immunology, the intestinal virome, and immune control of herpesviruses. Other talks on viral diseases highlighted the commonality of viruses that, like HIV, target mucosal sites for transmission and replication and emphasized the importance of cross-disciplinary studies in viral pathogenesis and vaccine development. Discussions also focused on T-cell and NK cell responses to HIV and on strategies to perturb the lymphoid tissue architecture to flush out residual, latently infected T cells from follicular sanctuary sites. Partnering with the immunology board was particularly appreciated, as the focus on mucosal and intestinal immunology in other virologic diseases is highly relevant to HIV. Dr. Hiroshi Kiyono (University of Tokyo, Japan) from the immunology board concluded the panel meeting with a remembrance and a moment of silence to commemorate Dr. Bonnie Mathieson, who passed away on January 8th, 2018. Dr. Mathieson was a program official at NIAID and the OAR for over 40 years, touching the lives of countless research scientists around the globe.

### 3.2. Acute Respiratory Infections (ARI) Panel

The ARI panel meeting took place on January 10th, 2018 and was chaired by Drs. Keigo Shibayama (National Institute of Infectious Diseases, Japan) and Florian Krammer (Icahn School of Medicine at Mount Sinai, U.S.A.), with secretariat Dr. Kristina T. Lu (NIAID, U.S.A.). The meeting sessions focused on 1) viral respiratory diseases, 2) respiratory syncytial virus (RSV), 3) Middle East Respiratory Syndrome Coronavirus (MERS-CoV), 4) coronavirus antiviral therapeutics, 5) transmission, pathogenesis, and immunity of influenza, and 6) influenza virus vaccines and inhibitors. The main objectives of the ARI panel meeting were to discuss recent research advances in viral acute respiratory infections and to establish collaborations and share resources amongst panel members and colleagues. The ARI panel meeting featured outstanding presentations of mostly novel, unpublished data followed by very active, constructive discussions and was deemed a huge success to be continued in 2019 for bacterial acute respiratory infections. Robust discussions and interactions occurred between the panel members and the audience, and several collaborations were further developed.

### 3.3. Cancer Panel

The cancer panel meeting was chaired by Drs. Edward L. Trimble (National Cancer Institute (NCI), U.S.A.) and Tohru Kiyono (National Cancer Center (NCC), Japan), and the secretariats Drs. Marie D. Ricciardone (NCI, U.S.A.) and Yukari Totsuka (NCC, Japan). The 1.5-day panel meeting convened with five sessions: 1) overview of hepatitis and liver cancer; 2) molecular biology of liver cancer; 3) non-surgical treatment of liver cancer; 4) early detection of liver cancer; and 5) summary and discussion of next steps. The panel meeting participants expressed strong interest in developing a research consortium to address prevention, screening, early detection, and management of liver cancer. They intend to form a working group of the National Cancer Institute (NCI), AMED, and academic researchers to develop research resources, including a clinical database and tissue biobank and foster stronger research collaborations on topics of mutual interest.

### 3.4. Hepatitis Panel

The hepatitis panel, “New Approaches to Hepatitis B Virus (HBV) Therapy” was chaired by Drs. Takaji Wakita (National Institute of Infectious Disease, Japan), Christopher Walker (Nationwide Children’s Hospital, U.S.A.), and Rajen Koshy (NIAID, U.S.A.; secretariat). The keynote lecture by Dr. Raymond Chung (Massachusetts General Hospital, U.S.A.) kicked off the two-day meeting with a discussion on the current understanding of a cure for chronic hepatitis B and the challenges for the complete elimination of HBV because of the stable and self-perpetuating replication intermediate of HBV DNA. The panel meeting included four sessions: 1) Chronic hepatitis B in Asia—scope of the problem and exploring new treatments; 2) markers of a functional cure for chronic hepatitis B; 3) preclinical animal models for testing HBV curative therapies; and 4) regulation of HBV replication and new targets for HBV therapy. In addition to the panelist presentations, posters were presented by early-stage investigators. The hepatitis panel meeting featured exceptional presentations, collaborative discussions, successful outcomes, and will be reconvened in 2020.

### 3.5. Viral Diseases Panel

The viral diseases panel was chaired by Drs. Richard Kuhn (Purdue University, U.S.A.) and Jiro Arikawa (Hokkaido University Graduate School of Medicine, Japan), with secretariat Dr. Eun Chung Park (NIAID, U.S.A.). The 1.5-day panel meeting focused on the following session topics: 1) hemorrhagic viruses including Ebola, Lassa, and Severe fever with thrombocytopenia syndrome virus (SFTSV), a highly pathogenic and recently emerged virus in Asian countries including Japan, Korea, and China; 2) enteric viruses including norovirus, rotavirus, poliovirus, and EV71, a virus causing hand and foot and mouth disease in Southeast Asian countries; 3) arboviruses including dengue, SFTSV, and chikungunya viruses; and 4) rabies virus and included aspects such as epidemiology, pathogenesis, structural biology, animal models, vaccines and antiviral developments, and human challenge models for dengue virus. There were significant presentations by junior/early-stage career scientists and discussions during the meeting were robust due to active participation and engaging discussions. Notable presentations from early-stage investigators selected from abstract submissions included a talk on a clinical trial of an investigational vaccine, called AGS-v, against mosquito saliva peptide. If proven effective, this vaccine can protect against mosquito-borne viral infections. Another presentation was on a broad-spectrum antiviral peptide that is shown to be active against Zika virus given post infection in an animal model. The viral diseases panel will reconvene in 2020.

## 4. Conclusions and Future Perspective

With more than 200 registrants, the 20th International Conference on Emerging Infectious Diseases in the Pacific Rim provided an opportunity for scientists and public health experts from 14 different countries and areas including Australia, China, Hong Kong, India, Iraq, Japan, Mongolia, Nepal, Singapore, Taiwan, Thailand, United Kingdom, U.S.A., and Vietnam to come together, interact, network, and discuss scientific developments and information on viral diseases of importance in the Asia-Pacific region and globally. The intent is that new or enhanced productive research collaborations will expand the knowledge and could lead to the prevention of infectious diseases and cancer. The program continues to evolve to engage more scientists from the region and specifically focuses on encouraging and fostering collaborations with young/early-stage career and female scientists from around the region. The 21st EID will convene in Hanoi, Vietnam from February 26–March 1, 2019. The local host was the Ministry of Health of Vietnam. The focus of the conference was on bacterial and parasitic diseases (for more information go to www.cvent.com/d/ytqb29). Furthermore, the program also continues to foster the research capacity of early-stage investigators and provide more opportunities for female investigators, through joint research call for proposals, funded by NIAID and AMED, on infectious diseases prevalent in Asian countries. The third round of joint collaborative research calls for proposals opened in October 2018, and the results will be announced in May 2019. In summary, the conference provided updates on the current status of infectious disease in the Pacific Rim region including information on new, emerging viruses like H7N9 avian influenza virus, SFTSV, CCHFV, and Zika, as well as pressing public health problems including hepatitis, dengue, and HIV.

## Figures and Tables

**Table 1:** Details of the USJCMSP International Conferences on EID in the Pacific Rim

	Date	City/Country	Topics	Local Chairs
**1st**	July 27–28, 1996	Kyoto, Japan	General Review	Tadao Shimao
**2nd**	March 6–8, 1997	Bangkok, Thailand	Enteric Infections Viral Infections	Wanpen Chaicumpa
**3rd**	March 28–30, 1998	Bali, Indonesia	Malaria, Tuberculosis, Hepatitis Infection and Malignancy	Pratiwi Sodermono
**4** **th**	March 2–4, 1999	Bangkok, Thailand	Parasitic Infections Influenza	Wanpen Chaicumpa
**5** **th**	January 7–9, 2000	Chennai, India	Tuberculosis and Leprosy HIV/AIDS	N.K. Ganguly
**6** **th**	January 13–15, 2001	Manila, Philippines	Bacterial Diarrheal Diseases Viral Zoonotic Diseases	Benjamin C. Vitasa Mario R. Festin
**7** **th**	October 31–November 1, 2002	Shanghai, China	Acute Respiratory Infections Parasitic Zoonoses	Linhua Tang Yumei Wen Zheng Feng
**8** **th**	December 11–12, 2003	Dhaka, Bangladesh	HIV/AIDS & Cholera and other Bacterial Enteric Infections	A.F.M. Sarwar Kamal
**9** **th**	December 10, 2004	Kyoto, Japan	Influenza	Yoshifumi Takeda
**10** **th**	November 16–17, 2005	Hanoi, Vietnam	HIV/AIDS and Tuberculosis	Nguyen Tran Hien
**11th**	November 16–18, 2006	Singapore	Avian Influenza	Chee Yam Cheng
**12th**	December 4–6, 2007	Haikou, China	Antimicrobial Resistance in Respiratory Infections	Yichen Lu
**13th**	April 6–9, 2009	Kolkata, India	Enteric Diseases	G. Balakrish Nair
**14th**	October 4–6, 2010	Penang, Malaysia	Diagnostics for Infectious Diseases	Asma Ismail, Rusli Ismail, M. Ravichandran
**15th**	March 11–13, 2013	Singapore	Vaccines and Protective Immunity (Viral Diseases)	Richard James Coker
**16th**	February 9–13, 2014	Dhaka, Bangladesh	Antimicrobial Drug Resistance in Bacterial and Parasitic Diseases	John D. Clemens
**17th**	January 26–27, 2015	Taipei, Taiwan	Emerging Viral Infectious Diseases	Chien-Jen Chen
**18th**	January 11–12, 2016	Bethesda, MD U.S.A.	Research Technologies/Innovations	NIH
**19th**	February 7–8, 2017	Seoul, South Korea	AMR of Bacterial and Parasitic Diseases	Jerome Kim
**20th**	January 8–9, 2018	Shenzhen, China	Pathogenesis and Protective Immunity of Viral Diseases	George Fu Gao
**21st**	February 26–March 1, 2019	Hanoi, Vietnam	Bacterial and Parasitic Diseases	Ministry of Health of Vietnam
**22nd**	February 2020	To be confirmed	Viral Diseases	
